# Hiding in the Shadows: Philip Morris and the Use of Third Parties to Oppose Ingredient Disclosure Regulations

**DOI:** 10.1371/journal.pone.0142032

**Published:** 2015-12-30

**Authors:** Clayton Velicer, Stanton A. Glantz

**Affiliations:** 1 Center for Tobacco Control Research and Education, University of California San Francisco, San Francisco, CA, 94143–1390, United States of America; 2 Center for Tobacco Control Research and Education, Cardiovascular Research Institute, Helen Diller Family Comprehensive Cancer Center, Department of Medicine, University of California San Francisco, San Francisco, CA, 94143–1390, United States of America; Utrecht University, NETHERLANDS

## Abstract

**Background:**

In 1996 Massachusetts proposed regulations that would require tobacco companies to disclose information about the ingredients in their products on a by-brand basis. This paper examines the strategies employed by Philip Morris to stop these regulations from being implemented.

**Methods and Finding:**

We used previously secret tobacco industry documents and published literature to examine the activities of the tobacco companies after the regulations were proposed. Philip Morris hired a public relations firm to establish a coalition that was instructed to oppose the regulations by linking them to other industrial sectors (the slippery slope) and stating they would damage the state's economy. Philip Morris also retained a polling firm to test the popularity of specific arguments against ingredient disclosure and developed a strategic plan for opposing similar regulations in Vermont.

**Conclusion:**

Tobacco companies have historically used third parties to form coalitions to oppose ingredient disclosure regulations. These coalitions have had success preventing regulations from being implemented after they are initially proposed by creating the appearance of local opposition. With countries around the world currently implementing ingredient disclosure regulations in the WHO Framework Convention on Tobacco, governments and regulatory agencies should be aware of the political strategies that the tobacco companies have used to create the impression of popular opposition to these measures.

## Introduction

Tobacco product ingredient disclosure regulation remains an important issue for both national and international regulatory agencies. In the United States, the US Food and Drug Administration (FDA) requires tobacco companies to submit information about their ingredients when applying for approval of new products or when claiming that a new product is ‘substantially equivalent’ to an existing product [1]. This information is kept secret from the general public on the grounds of trade secret protection. As of April 2015, 180 parties to the WHO Framework Convention on Tobacco Control (FCTC) had committed to requiring tobacco companies to disclose information about the ingredients in their products [2] and partial guidelines have been adopted for ingredient disclosure under the FCTC Articles 9 (regulation of the contents of tobacco products) and 10 (regulation of tobacco product disclosures) [3].

In 1986 Congress enacted the Federal Cigarette Labeling and Advertising Act (FCLAA) that required cigarette manufacturers to annually provide the U.S. Secretary of Health and Human Services (HHS) with "a list of the ingredients added to tobacco in the manufacture of cigarettes" (15 U.S.C Section 1355a). This disclosure did not provide any by-brand information and was prohibited from being disclosed to the public or other federal agencies. Without brand-by-brand information made available to scientists, regulators, and the general public, this form of disclosure made it impossible to research possible health impacts of different brands and prevented the consumer from having any information about the ingredients in the products they were smoking. In response to this lack of disclosure, Massachusetts [4] enacted ingredient disclosure legislation in 1996 that required the tobacco companies to disclose the ingredients in its' products on a brand-by-brand basis [5] to the Massachusetts Department of Public Health (MDPH), which was given the authority to make this information public. In November 1996 the MDPH held a public hearing to collect public testimony and written statements regarding the implementing regulations on January 30, 1997. Meanwhile, Brown and Williamson, Lorillard, RJ Reynolds and Philip Morris tobacco companies successfully fought this legislation in federal court [6,7]. At the same time the companies volunteered to conduct a study called the Massachusetts Benchmark Study that they inaccurately claimed would allow MDPH to estimate the levels of specific chemicals in the smoke of all brands on the market [8]. This analysis uses previously secret tobacco documents made available as part of litigation to research what strategies were used by Philip Morris in response to ingredient legislation proposed in Massachusetts and, then, Vermont. The company implemented public relations and political strategies to sway public and legislative opinion against ingredient disclosure policies, including use of third parties to form a coalition of businesses and trade associations to deliver the tobacco industry’s anti-disclosure message. Understanding these strategies can inform likely industry opposition to effective implementation of FDA regulation of tobacco products and implementation of FCTC Articles 9 and 10 [3].

## Methods

We used established methods for conducting research on tobacco industry documents [9,10]. Searches were conducted in the University of California San Francisco Legacy Tobacco Documents Library (LTDL: http://legacy.library.ucsf.edu) by the lead author (CV) between August 2014 and November 2014 for documents related to the tobacco companies' internal strategies for responding to state-level ingredient disclosure legislation, beginning with the search terms "ingredient disclosure and strategy" (2001 documents), "ingredient disclosure policy and state" (11,963 documents) and "Massachusetts Ingredients (3,073). Additional documents were found by reviewing adjacent documents (Bates numbers).

These searches identified tobacco industry documents detailing corporate strategies for dealing with proposed ingredient regulations in Massachusetts and Vermont. External organizations working with the companies were identified and subsequent searches were completed using their organization names (including "Chayet," a communications firm that worked with PM, and "Roper Starch," a polling company that frequently worked with the tobacco companies). We identified individuals working for Chayet using documents that listed their name and organization position. We completed subsequent searches using these names to identify additional organizations and individuals that had opposed ingredient disclosure.

As is standard procedure for tobacco documents research, data were not “extracted” from documents in the sense that information is extracted for a meta-analysis, but rather the information in the documents was used to prepare a history of the events described in the paper. We triangulated information from the available documents and other sources to validate and contextualize the information we found in the documents. As a practical matter, the results from the different sources were quite consistent.

S1 Text presented a much more detailed description of these methods.

## Results

### Hiring Consultants to Build a Coalition to Oppose Regulation

In October 1996, two months after ingredient disclosure legislation was enacted in Massachusetts, Philip Morris hired Chayet Communications Group to provide consulting services concerning ingredients disclosure, proposed statutes dealing with state Medicaid plans (likely mobilizing against emerging state lawsuits against the cigarette companies that sought to recover the costs to Medicaid of smoking-induced diseases) and proposed indoor smoking restrictions nationwide [11]. Chayet started working to establish a coalition in Massachusetts to mobilize political opposition against implementing the proposed regulations.

In November 1996, Neil Chayet, President of the Chayet Communications Group, wrote a letter to senior executives at Kraft Foods (then a Philip Morris subsidiary) stating the desire to form a coalition that would help put ingredient disclosure regulations “in a more appropriate light” with the public from the tobacco industry’s perspective [12]. (The Kraft CEO had previously opposed the disclosure of tobacco flavorings [13].) Chayet sought to win support for Philip Morris' efforts to oppose ingredient disclosure by expanding the issue to be larger than tobacco policy conflict and framing it as an issue that impacted other industrial sectors. Chayet tied the conflict to food and beverage producers, using the “slippery slope” argument that tobacco ingredient disclosure would affect future regulations: "it takes little imagination to contemplate the extension of the principle established by this statute to other products, most notably food" and that "food police" could use a similar approach to food "containing caffeine, fat, or whatever else zealous consumer organizations believe is harmful" [12].

In December 1996, Chayet wrote to the Kraft senior leadership summarizing their meeting [14], including an agreement that the food industry’s trade associations should be recruited to oppose the tobacco ingredient disclosure regulations [14]. A central strategy for doing so was to file an amicus brief in the pending lawsuit between the tobacco companies and the state of Massachusetts regarding ingredient disclosure. Kraft recommended to Chayet that the Grocery Manufacturers Association (GMA), a long-time ally of the tobacco companies [15], file an amicus brief. Following the December meeting with the Kraft, Chayet attended a luncheon with 15 representatives from local trade associations and businesses (Table 1) to discuss the importance of filing the amicus brief and developing testimony and statements to make against the ingredient regulations.

**Table 1:** Trade Associations that met with Chayet Communications in December 1996 [19].

Independent Oil Marketers of New England Associations (IOMA)
Best Petroleum
B&D Petroleum
Bursaw Oil
Mutual Oil
F.L. Roberts
Christy's Markets
New England Convenience Store Association (NECSA)
New England Service Station and Automotive Repair Association (NESSARA),
J Polep Distribution Services
McLane Northeast
Store 24
Massachusetts Petroleum Council

Although the GMA amicus brief was not ultimately filed, a draft appears in LTDL dated April 1997 [16]. The draft amicus brief closely aligned the tobacco ingredient disclosure with the food industry stating that GMA's members' food products "contain spices, flavors and colors that give each brand its distinctive taste and appearance” and emphasizes these are highly valuable and closely guarded trade secrets. In addition to arguing that federal law preempted state tobacco ingredient disclosure laws, the draft declared that a state law requiring food manufacturers to provide brand-specific information on spices, flavors and colors would conflict with Congress' purpose of protecting food ingredient trade secret information, upset the balance struck by Congress between public disclosure and protection of commerce, impose the state's product-disclosure policies on the entire nation and destroy the value of food manufacturers' trade secrets in every state and throughout the world [16].

On January 16, 1997, an employee working in the Philip Morris legal department forwarded a colleague a summary draft of how ingredient disclosure laws could impact other business and the value of intellectual property [17] with instruction to forward it to Chayet [18]. The draft explained the tobacco industry's current position and urged for action in the form of testimony or written statements at the upcoming hearing (January 30–31). This hearing was the final opportunity for all business concerned about intellectual property rights to urge the agency to issue final regulations which protect trade secrets to the greatest extent possible [17]. The draft also argued that the proposed regulations did not merely require the same level of disclosure for cigarettes as they did for food and beverages (as had been reported in the press) and that disclosure of all flavorings and trade secrets would be the same as exposing the formulae of Coke, Pepsi and Big Mac secret sauce [17].

One week later Chayet sent an email [19] with a list of "facts" to representatives from the trade organizations [20]. These "facts" consisted of the same arguments discussed by Chayet and Kraft that linked ingredient disclosure to other industries and claimed this kind of regulation was bad for the economy [20]. On January 27, an employee in Philip Morris legal wrote Chayet reporting that Philip Morris and the other tobacco companies had decided there was no "constructive purpose served by their appearing personally to testify at the MDPH hearings later this week" because "all indications are that rather than a balanced, unbiased forum to examine the proper scope of the proposed regulations, the hearings will be a "media circus" with the primary objective of bashing the tobacco industry so as to guarantee news headlines for the various government officials involved." Philip Morris urged Chayet to share this position with the coalition and advise that they could be submitted to hostile treatment and may wish to consider submitting their comments in writing to avoid this questioning [21]. Neither the trade organizations nor tobacco companies testified at the hearing; instead Chayet successfully mobilized the food trade organizations to submit written comments and a press release opposing the regulation [22].

On February 20, public comments were sent to the MDPH. Richard Salinsky, then president of the Best Petroleum Company based in Massachusetts and member of the Society of Independent Gasoline marketers of America (SIGMA), argued that the proposed regulations did not protect consumers because it could create the illusion of safer cigarettes [23]. Stalinsky said, "rather than having an effect of reducing risks to public health, [the regulations] have the opposite effect of increasing such risks by creating a false illusion that some brands of cigarettes are ‘more healthy’ than others." Salisnky also added that the disclosure regulation could create a threat to interstate commerce and would gravely injure the economic viability of SIGMA's members [23].

Representatives from the Independent Oil Marketers Association of New England (IOMA), New England Service Station and Automotive Repair Association (NESSARA), and Petroleum Marketers Association of America (PMAA) (Table 1) also submitted public comments to the MDPH arguing that ingredient disclosure and the accompanying risk to trade secrets would make tobacco companies stop selling cigarettes in Massachusetts and, so, harm local businesses [24,25,26]. They also argued that ingredient disclosure would create a black market for cigarettes that would hurt Massachusetts tax-paying business [26].

The next day the executive director of the New England Convenience Store Association (NESCA), issued a press release criticizing ingredient disclosure legislation that repeated the “bad for business” points in the fact sheet: "Our biggest fear is that if these regulations go into effect the tobacco companies will be forced to pull their products from the shelves of our stores in Massachusetts to protect their trade secret rights" [20]. The release continued, "there are many people that would applaud that action but we are not among them because our members would be devastated by this loss of revenue and be forced out of business" [27]. NESCA was a previous ally of the tobacco companies in Massachusetts where they had helped promote the Tobacco Institute's “It's the Law Program” to help prevent more strict youth access to tobacco regulations[28] and were paid $12,500 for their effort [29].

### Polling the Public

In March 1997, Philip Morris hired Roper Starch, a polling firm frequently used by the tobacco industry [29,30,31], to conduct interviews with a nationally representative sample of 2000 adults on ingredient disclosure [32]. The poll found strong support for tobacco product ingredient disclosure (74% of smokers, 73% of non-smokers), but less support for consumers' right to know the exact amount of each ingredient (46% and 42%) and even less for information on specific brand formulas (35% to 32%) [32]. Roper Starch also asked about regulations in other kinds of products, specifically food, household cleaning products, paint, alcoholic beverages and soda. A minority of smokers (38%) and nonsmokers (35%) believed there was not enough regulation on food and product labeling which was lower than the results for the regulation of tobacco sales and marketing: (smokers 56% and nonsmokers 58%). These findings may have provided important intelligence that led the Philip Morris-hired communications group to specifically mention food (Big Macs) as an area that ingredient legislation could impact in their "fact sheet." Roper Starch found 68% of both smokers and non-smokers wanting to know the exact brand formula for tobacco products compared to 40% of smokers and 38% of non-smokers wanting the information for soda products [32].

Roper Starch also polled which different arguments were viewed as being the strongest in opposition of ingredient regulation (Table 2). These arguments were focused on government overreach (e.g., government overstepping its' bounds in regulations) or regulations in one industry (tobacco) impacting future regulations in other industries (food, alcohol).

**Table 2:** Poll by Roper Starch conducted for Philip Morris [32] (March 1977).

Argument	Agree that Very or Fairly Good Argument	
	Smokers	Nonsmokers
It doesn't make any sense for 50 states to have 50 different sets of rules governing the disclosure of tobacco brand formulas.	75%	68%
Tobacco companies should have the same right as any other company to keep competitors from seeing or copying their brand formulas.	71%	61%
More product disclosure regulation will mean creating another government bureaucracy to make sure the information is accurate with taxpayers footing the bill.	66%	59%
Since the federal government already reviews every tobacco product ingredient for safety, there is no reason for the states to do the same thing.	62%	55%

In the end, the cigarette companies won in court and the effort for ingredient disclosure in Massachusetts died [33].

### Vermont

In February 1997, a bill was introduced in the Vermont Legislature to require tobacco companies to disclose the "identity of any additive other than tobacco, water or reconstituted tobacco sheet made wholly from tobacco and the weight, measure or numerical count of each additive" [34] and gave the Vermont Department of Public Health the authority to release the information to the public if it was deemed to be in the interest of public health. Philip Morris prepared a political strategy for fighting ingredient disclosure in Vermont [35] that mirrored its Massachusetts activities in 1996 and 1997 and provides a more detailed description of its two-phase approach to defeat the bill: (1) building third party alliances (the “educational outreach”), and (2) legislative phase.

As part of the education outreach Philip Morris planned to engage the Vermont Chamber of Commerce, Vermont Grocers, Vermont Lodging and Hospitality Association and the Vermont National Federation of Independent Businesses through a series of briefings to explain why brand ingredient disclosure is bad for local business [35]. The plan states "In all of our efforts we have a single communications message: State legislation requiring disclosure of tobacco product ingredients is preempted by federal law. Any attempt to legislate this at the state level represents a slippery slope that could lead to the forced disclosure of trade secrets and recipes for a variety of products including ice cream to pancake syrup" [35]. (Ice cream and maple syrup are major products produced in Vermont.) Philip Morris suggested creating an op-ed to be submitted by the Vermont Chamber of Commerce and providing letters to small business owners and third party executive directors to submit to their local newspapers. Philip Morris also planned polling that they could release showing overwhelming opposition to brand ingredient disclosure.

The second "legislative process phase" included mobilizing business owners, managers and executives to send letters to their legislators. Philip Morris also planned to try and mobilize the general public with materials placed at the point of sale, including postcards placed at restaurant counters for customers to take, sign, fill in their name and address and return to the restaurant manager. The postcards read, "As a Vermont consumer I believe state government is going way to far buy [sic] singling out one product that happens to be in disfavor, and requiring it's makers to share their recipes and trade secrets. When government starts wading into these-type of issues, it is getting too big and our freedoms are being lost" [35].

The legislative phase also anticipated utilizing proactive programs on youth issues to counter negative publicity generated from the ingredients disclosure debate including becoming "hyper-aggressive" in execution of Philip Morris’ Action Against Access (AAA). The AAA was a program nominally designed by Phillip Morris to prevent youth access to tobacco through a series of voluntary steps including placing minimum age language on packages, training vendors on sales to minors and providing materials on asking for proof of age identification [36,37]. This full plan does not appear to have been put in to effect because the Vermont bill died after being sent to committee, but provides evidence that Philip Morris planned to employ similar strategies to fight ingredient disclosure to those used in Massachusetts.

## Discussion

The use of trade associations and business by Philip Morris in Massachusetts (and proposed plans in Vermont) serve as an example of the kind of strategies that tobacco companies and large businesses can employ to fight regulations that are likely to negatively impact their business. These strategies can be employed after regulations are formally proposed to prevent them from actually being implemented by creating the appearance of localized and broad-based opposition. The tobacco companies have previously used this strategy to oppose regulations, including opposing tax increases on cigarettes [38,39], smokefree laws and policies [40,41,42,43,44,45,46], and health care legislation [47]. Through these efforts, the tobacco companies increased the number of involved parties in the conflicts by framing the issue as part of a larger conflict that impacts other industries. Philip Morris executed this strategy in response to the ingredient regulations by using their power as a large corporation to poll the public to see which industries could most effectively be brought into the conflict.

The tobacco industry has attempted to expand conflicts in response to previous proposed regulations. In 1989 the tobacco industry planned to oppose Congressional legislation focused on preventing youth from seeing cigarette advertising [48] (H.R.1250, the Protect our Children from Cigarettes Act) by "make[ing] the slippery slope real." The industry planned to expand the conflict by arguing that restriction on tobacco advertisements would lead to restrictions on other products. They planned to build a coalition of industries that were currently under fire for their advertising (specifically mentioning Maddona and Pepsi) and brainstormed bringing in additional industries by developing advertisements that portrayed their products as dangerous (specifically mentioning a car advertisement showing a car driving over 55mph or under unsafe conditions [49]). In 1994, after the FDA announced it would consider regulating nicotine as a drug, RJ Reynolds launched "Project Breakthrough" a campaign to convince Americans that anti-smoking advocates wanted cigarettes to be completely prohibited [50]. The campaign was aimed at spreading the fear that other products could be made illegal including alcohol, beef, pork, private property, logging, fur, cholesterol and motorcycles [50].

Another strategy in response to ingredient disclosure was Philip Morris' Vermont plan to "change the subject” and counter negative publicity by focusing on industry youth access programs [35]. In addition to being ineffective, these programs serve to shift attention away from the tobacco industry's behavior and onto programs in which the industry can be an active partner [44,51,52,53,54]. This strategy has three main benefits for tobacco companies: (1) Promoting 'positive' policies (like youth access programs) promotes the perception that the tobacco companies are socially responsible businesses whose interests deserve serious consideration by legislators [44,51,52,53,54,55,56,57,58,59] (2) Aiming to shape the policy agenda by highlighting where public health intervention should be targeted, such as in the tobacco companies’ “accommodation program” [42,60,61,62] in response to the idea of restricting smoking indoors. This accommodation program was designed to "establish a framework for self‐regulation that precludes the need for government intervention" [63]. As part of accommodation, Philip Morris developed surveys with the International Hotel Association (IHA) that showed 90% of IHA members felt "smokers are important to their business" and 89% felt it was important to accommodate both smokers and non-smokers while also promoting the concept of ventilation as effective [63]. (3) Through the efforts of point 1 and 2 changing the subject accomplishes a third goal by suggesting the industry's opposition to public health measures is not driven purely by commercial imperatives. When promoting youth access programs, the tobacco companies are promoting a practice that would in theory reduce some of their sales (teenagers purchasing cigarettes without proper evidence of age) because it is the "right thing to do." In the example of accommodation, they are not opposing indoor smoking because it is bad for business, but because it is important that businesses be able to provide an environment that is enjoyable to all their customers, rather than just smokers or non-smokers. In other words, the accommodation is a program that works for everyone and is the right business decision for the hospitality industry rather than the cigarette company itself.

Philip Morris showed another advantage of large corporations in opposing regulations with their use of polling to try and influence public opinion. The polling conducted by Roper Starch compared interest in ingredient disclosure for tobacco products compared to other industries. This information was then used to inform requests for public comment that created the appearance of localized opposition. In Massachusetts, Philip Morris urged comments against the disclosure regulations claiming that requiring ingredient disclosure for cigarettes would lead to similar demands for the soda industry and the Big Mac Secret Sauce ingredients [17]. (Roper Starch had previously found the public had less interest in learning about the exact brand formulas in soda and fast food products compared to tobacco [32]).

These strategies and the difficulties implementing ingredient disclosure regulations in Massachusetts are relevant to informing the for Conference of the Parties as it develops implementing guidelines and protocols for FCTC Articles 9 and 10 [3]. In the 2014 the Convention Secretariat reported that implementation of Article 9 (regulation of the contents of tobacco products) and Article 10 (regulation of tobacco product disclosures) were in the middle range of implementation compared to implementation of all FCTC articles [64, 65]. For Article 10, the progress report states "several parties that have already developed relevant regulations report on the shortage of independent (i.e. not run or influenced by the tobacco industry) testing facilities or laboratories and/or lack of access to such testing facilities; Parties also refer to recent legal challenges filed by the tobacco industry in this area” [64]. As countries work to implement FCTC Articles 9 and 10 (and the FCTC general), they need to be cognizant of these ways in which the industry tries to build and use alliances to oppose regulation, including the use of third parties. This experience also points to the importance of requiring disclosure of interests and potential conflicts in all public submissions, including from groups that include any businesses or membership. Such a requirement could facilitate transparency linking these groups back to the industry that was orchestrating them and be helpful in estimating the validity of public comments made during legislative and regulatory processes.

### Limitations

Since these findings are based on documents turned over by the tobacco companies, discussions and strategies that occurred offline (such as in person meetings without notes being taken or over the phone) are not available to us. Additionally, documents that contain privileged or confidential information could have been withheld from public access. It is possible that with access to additional information we would be able to describe in even greater details some of the strategies employed by Philip Morris to fight ingredient disclosure laws.

### Conclusion

The strategies employed by Philip Morris in Massachusetts and proposed plans in Vermont in response to ingredient regulation serve as an excellent example of how tobacco companies, and large industries as a whole, may respond to regulation. The strategies of expanding the conflict, changing the subject and conducting polling to determine message framing are all strategies that can be used to increase the appearance of localized and broad based opposition. Philip Morris' use of a third party coalition in Massachusetts should be carefully considered and serve as another reminder that "opposition" to such regulations is often manufactured by the tobacco companies. As new organizations emerge voicing opposition to FCTC Articles 9 and 10 or in future ingredient regulations in the United States, these organizations and individuals should be carefully evaluated to determine their links to tobacco companies in order to challenge the the impression of popular opposition to these measures.

## Supporting Information

S1 Text Additional Details on Methods. (PDF)
