# Supplementary material for: Hiding in the Shadows: Philip Morris and the Use of Third Parties to Oppose Ingredient Disclosure Regulations
Source: PLoS One. 2015 Dec 30;10(12):e0142032. doi: 10.1371/journal.pone.0142032 (PMC4696670; doi:10.1371/journal.pone.0142032)
Supplement: S1 Text — (PDF) [file pone.0142032.s001.pdf]

SUPPLEMENTAL TEXT S1. ADDITIONAL DETAILS ON METHODS

Hiding in the Shadows: Philip Morris and the Use of Third Parties to Oppose Ingredient Disclosure Regulations

Clayton Velicer, MPH
Stanton Glantz, PhD

As noted in the main text, this paper uses well-established methods for tobacco industry documents research that have been described in detail in methods papers (Malone RE, Balbach ED. Tobacco industry documents: Treasure trove or quagmire? *Tob Control*. 2000 Sep;9(3):334-8 and Anderson SJ, McCandless PM, Klausner K, Taketa R, Yerger VB. Tobacco documents research methodology. *Tob Control*. 2011 May;20 Suppl 2:ii8-11). This supplemental text provides more details on methods for readers who are not familiar with tobacco industry documents research (presented in the context of this paper).

If every document retrieved was not used in your analysis, what was the process for selecting the documents (screening, eligibility) and who conducted this procedure? How many documents were excluded from the analysis?

When searching for a specific phase, for example, "Massachusetts Ingredients" there are many results retrieved (3,073 for this phrase). In the beginning of the search all of these documents are viewed. Documents are set aside for further review if they include any information relevant to the study. For example, communications between the Massachusetts Department of Public Health and tobacco company employees or between tobacco employees and third party organizations related to ingredient disclosure would be considered relevant and included in the analysis if they contain unique information not contained in other documents.

Some documents mention Massachusetts Ingredient Disclosure, but provide no information related to the tobacco company's strategy for ingredient disclosure. In December, 1997 the tobacco company Liggett decided to disclose information about the ingredients in their product (http://industrydocuments.library.ucsf.edu/tobacco/docs/#id=ynkk0068). While related to the event of ingredient disclosure, this event did not mention any of the strategies employed by Philip Morris or other companies regarding disclosure and was not included in our analysis.
.

Another problem with tracking the number of "excluded documents" is that through the process of documents being made available to the public many duplicate copies of documents are created. This occurs for a variety of reasons, but the most basic example would be that if one scientist sends another scientist an email and two scientist are also cc'd there would immediately be 4 copies of the email in the legacy library. If this email was then forwarded to another scientist, an additional copy would be created. Thus, mentioning that 1 of the 5 emails was included in the analysis would provide no added value and could serve to confuse the reader.

Because of such situations, the number of documents that were excluded from the analysis is not a figure that is been reported in papers based on tobacco industry document research because it does not provide any useful information to the reader.

The searches were conducted by the first author, Clayton Velicer, who, at the time this paper was written, had over 2 years experience conducting analysis with the documents.

How were data extracted from the documents and were there any extra steps taken to confirm the information?

Data were not "extracted" from documents in the sense that information is extracted for a meta-analysis, but rather the information in the documents was used to prepare a history of the events described in the paper. Similar methods have been used in 907 peer reviewed papers and other publications based on the Legacy Tobacco Documents Library (http://www.refworks.com/refworks2/?site=044531177905600000%2fRWWS6A586201%2fTobaccoDocumentsBibliography).

The Methods section states, "Additional documents were found by reviewing adjacent documents (Bates numbers)." Was every adjacent document included in the search? If not, what method was used for determining the inclusion of adjacent documents?

Not every adjacent document was included. Related to the answer for question number 1, adjacent documents were included in the analysis if they provided any additional or relevant information that was not available in other documents. If the adjacent documents contained duplicate information or were not related to the topic of analysis, they were not included.

The Methods section states,"…and searching for individuals identified as employees at the third party organizations or Philip Morris."

a. What names were used?

The names were people identified in the documents that we located beginning with key word searches.

Here is an example of how a snowball search works for individuals: After searching for the phrase "Massachusetts and Ingredients" a document was found featuring Neil Chayet communicating with Cal Collier about ingredient disclosure in Massachusetts. CC'd on this list were two additional individuals Paul Petrucilli and Mark Berlind. All of these individuals were then subsequently searched for with the phrases "Massachusetts" and "ingredient disclosure." If these subsequent individuals were found to have communications with additional people regarding ingredient disclosure in Massachusetts these names would also be subsequently searched.

However, the focus of on search would be on the content of the document rather than every name that is included on an email. In many cases individuals are included on an email for one specific topic (sometimes as legal advisors or administrative assistants) but are not relevant

to the larger picture of ingredient disclosure. After searching for an individual's name reveals they are only serving in an administrative capacity and no new content is discovered using their name they are not included further in the analysis.

While documents that can be potentially useful are stored for further evaluation, cold documents are not carefully tracked because this has not historically served any purpose to the author or reader. Sharing with the reader the amount of people searched that did not relate to the topic would be confusing.

b. How did we determine whether these individuals were employees of Chayet, Roper Starch, or Philip Morris?

Many of the documents, particularly letters, list an individual's full name and title at an organization. If a document does not list an individual's affiliation or place of employment a subsequent search of their name is completed until a document is found that identifies their place of employment.

c. How did we determine which employees to include in our search?

As explained in part a, any name that was discovered in a document related to the topic of ingredient disclosure in Massachusetts would have their name subsequently searched for in the Legacy Tobacco Documents Library (LTDL; http://legacy.library.ucsf.edu). A number of names were identified in the results for broad search terms like "Massachusetts and ingredients." Subsequent names to search were found by searching the names from these initial findings.

5**. How were the archived news stories on NewsBank retrieved and reviewed, if different from the LTDL strategy?**

The strategy was the same as for the LTDL searches.

6. What methods were used to synthesize findings from disparate sources?

Following standard practices for tobacco documents research, we use *triangulation* (Maanen J, Dabbs J, Faulkner R, editors. Varieties of Qualitative Research. Beverly Hills, CA: SAGE Publications; 1982; Patton M. Qualitative Research and Evaluation Methods. 3rd, editor. Thousand Oaks, CA: SAGE Publications; 2002) among the available documents and with other sources to validate and contextualize the information we find in the documents. (Triangulation, which examines consistency from independent sources, is standard for this type of qualitative research.) As a practical matter, the results from the different sources were quite consistent.

7. Are there any limitations of the methods that could affect the outcome of the study?

Since these findings are based on documents turned over by the tobacco companies as a result of discovery in litigation. Discussions and strategies that occurred offline (such as in person meetings without notes being taken or over the phone) are not available to us. As noted in the Limitations section of the main paper, documents that contain privileged or confidential

information can be blocked from being made available to the public. It is possible that with access to additional information we would be able to describe in even greater details some of the strategies employed by Philip Morris to fight ingredient disclosure.
